# Investigation of patterned and non-patterned poly(2,6-dimethyl 1,4-phenylene) oxide based anion exchange membranes for enhanced desalination and power generation in a microbial desalination cell

**DOI:** 10.1016/j.ssi.2017.11.004

**Published:** 2018-01

**Authors:** Francisco Lopez Moruno, Juan E. Rubio, Carlo Santoro, Plamen Atanassov, José M. Cerrato, Christopher G. Arges

**Affiliations:** a Department of Civil Engineering, University of New Mexico, Albuquerque, NM, USA; b Center Micro-Engineered Materials (CMEM), Department of Chemical and Biological Engineering, University of New Mexico, Albuquerque, NM, USA; c Cain Department of Chemical Engineering, Louisiana State University, Baton Rouge, LA 70803, USA

**Keywords:** Microbial desalination cells, Anion exchange membranes, Desalination, Power electricity generation, Transport phenomena

## Abstract

Quaternary ammonium poly(2,6-dimethyl 1,4-phenylene oxide) (QAPPO) anion exchange membranes (AEMs) with topographically patterned surfaces were assessed in a microbial desalination cell (MDC) system. The MDC results with these QAPPO AEMs were benchmarked against a commercially available AEM. The MDC with the non-patterned QAPPO AEM (Q1) displayed the best desalination rate (a reduction of salinity by 53 ± 2.7%) and power generation (189 ± 5 mW m^− 2^) when compared against the commercially available AEM and the patterned AEMs. The enhanced performance with the Q1 AEM was attributed to its higher ionic conductivity and smaller thickness leading to a reduced area specific resistance. It is important to note that Real Pacific Ocean seawater and activated sludge were used into the desalination chamber and anode chamber respectively for the MDC – which mimicked realistic conditions. Although the non-patterned QAPPO AEM displayed better performance over the patterned QAPPO AEMs, it was observed that the anodic overpotential was smaller when the MDCs featured QAPPO AEMs with larger lateral feature sizes. The results from this study have important implications for the continuous improvements necessary for developing cheaper and better performing membranes in order to optimize the MDC.

## Highlights

• Novel anion exchange membranes were used to develop effective MDCs.
• AEMs with different topographical patterns were investigated.
• Maximum power achievement was 189 ± 5 mW m^− 2^.
• Maximum desalination achieved was 53 ± 2.7% over 3 days.
• QAPPO AEM membrane outperformed commercial membrane.

## 1 Introduction

Stress on water availability and quality is a worldwide concern, particularly in semi-arid regions [1]. Even though potable water and water used in agriculture and energy production are stressed in some areas of the world, water as a general resource is not in short supply. There is plenty of water available, but 97% of it is mixed with salt rendering it unusable [2], [3]. Remediating this process requires water treatment and water desalination, which has been adopted using numerous technical processes over the past 30 years. The majority of those treatment processes is energy intensive and therefore is widely operated mainly in developed countries that typically have low energy costs. Particularly, Middle East countries, in which water is very scarce and only in salty form, are accelerating grass roots construction of large desalination plants to obtain drinking water for civil use [4]. However, construction of large desalination plants for the rest of the globe has been slow because of these plants' large capital costs (mainly ascribed to the membranes), high energy costs, and environmental concerns [5], [6], [7].

Distillation and reverse osmosis are the most common water desalination processes. Distillation, the most popular technology, accounts for 60% of water desalination plants in the world, while the second most adopted technology is reverse osmosis with a 40% [8], [9]. Distillation utilizes heat to phase change the water (from liquid to gas and then back to liquid) in order to separate the water from the salt [10], [11]. Therefore, a heating source is needed and it is usually obtained from exhausted heat from power plant in order to minimize the energy cost required. The negative aspect is that that energy could be used further to generate electricity in a combined cycle, decreasing the overall efficiency of the power plant [10], [11]. Reverse osmosis also is a very energy extensive technique based on the application of high pressure in order to overcome the natural osmotic pressure and separate the water from the ions through semipermeable membranes [8], [9], [12]. Plus, the membranes in reverse osmosis are costly and need to be replaced periodically as they are prone to fouling [13]. Reverse osmosis has a smaller energy footprint compared to distillation, but maintenance costs associated with membrane replacement make it a costly proposition [6], [7]. Faced with these problems, a diverse set of new technologies are emerging to complement, or supplant, these current technologies to lower the energy footprint for water desalination while having competitive capital costs and low restate foot prints while being highly automated and robust.

One alternative desalination technology under consideration since 2009 is a microbial desalination cell (MDC), a type of bio-electrochemical cell [14], [15], [16], [17]. MDC is a promising technology with trigenerative aspects such as wastewater treatment, electricity generation and water desalination. A MDC is a galvanic, self-sustainable bioelectrochemical system (BES), in which electroactive bacteria are able to convert organics and pollutants at the anode into electrical energy through the biological and electrochemical reactions [18]. At the cathode, oxygen is electrochemically reduced to complete the circuit [14], [15], [16], [17], [18]. This system has a central chamber separated from the other two chambers (anode and cathode chamber) by an anion and cation exchange membrane. The selective membranes allow the transfer of ions from the salty water (mainly Na^+^ and Cl^−^) to the other chambers. A unique feature of the MDC is that it can reduce the salinity content in the central chamber, while co-currently producing electrical energy through electrochemical oxidation of organics and pollutants [14], [15], [16], [17], [18].

Despite the innovative and promising aspects regarding MDCs, there are existing issues with this technology that require improvement. The different, and diverse, elements in MDC can vary significantly altering the desired objectives of the technology (e.g., power output and desalination amount). A pilot scale MDC was scaled up to 100 L [19], but the scaled MDC revealed that the technology requires significant resolution to a plethora of problems to make it commercially lucrative. In particular, the principal problems related with MDCs are: low electrochemical performances, poor chemical oxygen demand (COD) degradation, and unsatisfactory desalination rates [20], [21], [22], [23], [24], [25], [26], [27]. Several investigations have aimed to improve the MDC system by optimizing the design of elements and employing different membrane and electrode materials. The ultimate goal is to enhance energy recovery and extract higher desalination rates [20], [21], [22], [23], [24], [25], [26], [27]. For example, the cathodic reaction for the MDC has been investigated with either a potassium ferricyanide or oxygen reductant species. Oxygen was the more appropriate species due to its: i) its natural availability; ii) low cost; and iii) has a high reduction potential [28]. Other different approaches were pursued to enhance the system as for example the utilization of biocathodes, bipolar membranes, capacitive features, or recirculation [15], [16]. In parallel, low content of easily degradable chemical oxygen demand (COD) present in the wastewater that is used as fuel from the microorganisms at the anode, negatively affect the anodic electro-kinetics [15], [16]. Greater improvements related to anode and cathode, as well as the membranes, are important priorities to enhance the sluggish electrochemical performances.

Microbial desalination cell technology has been investigated since the past decade. For example, the initial studies of X. Cao in 2009 [14], using AEM (DF120, Tianwei Membrane) and CEM (Ultrex CMI-7000, Membranes International) obtained promising results. Investigations conducted by other research groups have also used mostly commercially available membrane from Membranes International INC. New Jersey, USA (AEM AMI-7000 and CEM CMI-7000) [21], [29], [30]. However, the effect of using different AEMs to improve the performances and the desalination rate has been overlooked. This serves as a motivation for the investigation of novel AEMs in this study which could be a feasible way to further enhance the performances of MDCs, especially in terms of power generation and reduction in salt content.

The objective of this study is to investigate the electrochemical performance of a MDC in terms of power density and desalination rate utilizing different quaternary ammonium poly(2,6-dimethyl 1,4-phenylene oxide) (QAPPO) AEMs with non-patterned and patterned topographical features. The MDC cell that examined the different AEMs utilized a common commercial cation exchange membrane, while examining three different solutions: i) activated sludge; ii) Pacific Ocean seawater; and iii) 10 mM potassium phosphate buffer (K-PB). Operating parameters, such as pH and solution conductivity, were monitored during the experiments to estimate the desalination rate. The operating conditions and desired outputs, power density and desalination rate, of the MDC with different AEMs were benchmarked against commercial AEMs.

## 2 Materials and methods

### 2.1 Electrodes used in microbial desalination cells

The anode electrodes used during the experimentation were carbon brushes made by carbon fibers wrapped on a titanium core (Millrose, USA). The anode had a cylindrical shape with a 3 cm diameter and 3 cm length. Initially, the anodes were taken from an existing microbial fuel cell with anodes already well colonized with electroactive bacteria and therefore ready to work [31], [32].

The cathode electrodes were air-breathing cathodes designed for oxygen reduction reaction (ORR). This cathode configuration was presented previously [33], [34], [35], [36]. Particularly, the cathodes were prepared by grinding activated carbon (AC), carbon black (CB) and polytetrafluoroethylene (PTFE). After AC/CB/PTFE in ratio of 8:1:2 were mixed, the obtained black powder was inserted into a pellet die and pressed using a hydraulic press on a stainless-steel mesh used as current collector. AC/CB/PTFE had a loading of 40 mg cm^− 2^. The cathode geometric area exposed to the electrolyte was 7 cm^2^ [31], [32]. Particularly, the catalytic side was exposed directly to the liquid electrolyte, while the metallic mesh was facing the ambient atmosphere. New cathodes were used at each cycle, although the cathodes used did not suffer any kind of degradation during the cycles, not showing any leakage of biofouling over their surfaces.

### 2.2 Membrane materials: fabrication and characterization

Anion and cation exchange membrane were used to physically separate the desalination chamber, positioned between the anode and the cathode chamber respectively. The cation exchange membrane (CEM) utilized during this experiment was a commercial cation exchange membrane, CSO, 100 μm, AGC Engineering CO., LTD, Japan. PPO (Mn: 20 k; PDI ~ 2.5) was sourced from Polysciences Inc. All other chemicals used to make PPO were sourced from VWR except 2,2′-Azobis(2-methylpropionitrile) (AIBN – free radical initiator – 99%, recrystallized), which was attained from Sigma-Aldrich.

Freestanding AEMs composed of poly(2,6-dimethyl 1,4-phenylene oxide) (PPO) with quaternary benzyl trimethylammonium chloride moieties were synthesized as reported in the literature [37]. The synthesis procedure is briefly summarized here: PPO was dissolved in chlorobenzene (8 wt%) at room temperature. The dissolved polymer was transferred to a round bottom flask with an egg-shaped stir bar. *N*-bromosuccinimide (NBS) was added (0.7:1 M ratio to PPO repeat unit). The reaction solution was heated to 130 °C. The free radical initiator AIBN was added (2 wt% to the amount of PPO dissolved). After reacting the solution for 18 h, the reaction solution was cooled to room temperature and was precipitated in methanol (5:1 volume ratio). The collected polymer was then dissolved in chloroform and precipitated in the methanol (5:1 volume ratio) to remove impurities. Then, brominated PPO (BrPPO) was dissolved in *n*-methyl-2-pyrrolidone to make a 5 wt% solution. 40 wt% of trimethylamine water was added in limiting reagent (0.5 trimethylamine to bromomethyl group). (Note: Trimethylamine was added in limiting reagent to prevent excess swelling of the QAPPO AEM in a fully flooded cell. The unreacted bromomethyl groups self-crosslinked to reinforce the membrane's mechanical properties [38]). The reaction was stirred at room temperature for 48 h and then drop casted onto substrates to prepare non-patterned and patterned AEMs. The AEMs were then removed from the substrates by immersing in deionized water and peeling the membranes off the substrate. 50 μm thick QAPPO AEMs were attained. The QAPPO AEMs were ion-exchanged from the bromide counterion to the chloride counterion by immersion in 1 M sodium chloride (NaCl) overnight followed by immersion and excess rinse with deionized water to remove residual salt ions.

The QAPPO AEMs with different periodic, topographical patterns were prepared by drop casting the dissolved QAPPO solution in NMP on to micropatterned poly(dimethyl siloxane) (PDMS) molds that were prepared through conventional soft lithography. The different lateral feature sizes of the patterned QAPPO AEMs were: 20 (Q2), 33 (Q3), 40 (Q4), and 80 (Q5) μm. The non-patterned QAPPO AEM (Q1) was drop casted onto a flat glass substrate.

The conversion of the base polymer, PPO, to BrPPO was confirmed via ^1^H NMR spectroscopy using deuterated chloroform (CDCl_3_) solvent that contained tetramethylsilane (TMS) as an internal standard. The NMR spectrometer was 400 MHz Bruker instrument. The amount of bromine added to the PPO backbone was determined by integrating the ^1^H NMR spectra according to the literature [37]. The ionic conductivity of the non-patterned QAPPO AEMs was determined by electrochemical impedance spectroscopy (EIS) using a 4-point platinum conductivity probe. EIS, in galvanostat mode, was performed with a 2 mA amplitude in the frequency range of 100,000 Hz to 0.1 Hz. The in-plane resistance was determined from the Bode plot, where the resistance value had a phase angle value of zero, and was used in Eq. (1) to determine the in-plane ionic conductivity (σ).

(1) $$\sigma =\frac{L}{R\times t\times w}$$

where σ was the in-plane conductivity, R was the in-plane membrane resistance, t was the membrane thickness (fully hydrated membrane) and w was the membrane width (fully hydrated membrane).

As check control and comparison for this study, an AMI-7001S AEM (from Membranes International INC. New Jersey, USA) was also assessed in MDC cell. The through plane resistance for this membrane and thickness, as stated by the manufacturer, is: < 40 Ω-cm^2^ and 450 μm [39].

### 2.3 Set up and operating conditions

**Fig. 1:**
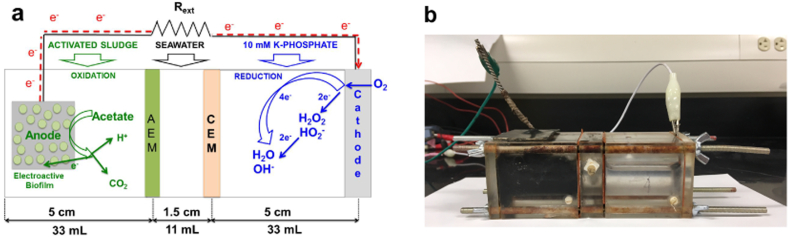
Microbial desalination cell (MDC) setup used for this study (a) MDC schematic; and (b) picture of operating MDC.

The system used for this study consisted in a MDC having three separated chambers (anodic, desalination and cathodic chamber), an anode electrode (immersed into the anodic chamber), an air-breathing cathode, and two exchange membranes separating the three chambers. This setup is illustrated in Fig. 1. Anodic and cathodic chambers had a volume of 33 mL, instead the desalination chamber had a volume of 11 mL [31], [32]. The anode chamber was filled with a 33 mL solution of activated sludge taken from Albuquerque Southeast Water Reclamation Facility. The initial pH of the sludge was 7.8 it had an initial conductivity of 2.1 mS cm^− 1^. The solution was fully replenished for every cycle. The central chamber of the system, named desalination chamber, was filled with 11 mL of real seawater (51.4 mS cm^− 1^) collected from the Pacific Ocean in Solana Beach–CA-USA. An anion exchange membrane (AEM) separates the anode chamber from the desalination chamber. The cation exchange membrane (CEM) separates the cathode chamber from the desalination chamber. The data recorded at the study was based on 3 days cycles for each cell, doing a total of 3 cycles for each combination of membranes (triplicate results). MDCs experiments were run always using the same operating conditions.

### 2.4 Measurements

#### 2.4.1 Solution conductivity and pH

Solution conductivity and pH were recorded initially and every 24 h during each cycle. The pH was measured using an Omega PHB-600R (Omega Engineering Inc., Norwalk, CT, USA). Solution conductivity was determined using an Orion Star 112 Conductivity Meter (ThermoFisher Scientific, Waltham, MA, USA). Both instruments were calibrated periodically (every cycle) to have consistent data.

#### 2.4.2 Electrochemistry

After filling the chambers with the previously described solutions, anode and cathode were connected to an external resistance of 470 Ω. Linear Sweep Voltammetries (LSVs) were carried out in order to obtain the overall polarization curves and the power curves. Two potentiostats Gamry Reference 600 + (Gamry Instruments, PA, USA) were used during the experiment. In the first one, two electrode configuration was used with anode connected to the counter electrode, cathode to the working electrode and the reference channel short circuited with the counter electrode. LSV was run between open circuit voltage (OCV) and 0 mV at a scan rate of 0.2 mV^− 1^. The other channel was used to measure the potential of the cathode during the LSV (counter electrode short circuited with the reference channel and connected to a Ag/AgCl (3 M KCl) and the cathode connected to the working electrode). The reference electrode was a Ag/AgCl (3 M KCl) that was placed in the desalination chamber. V-I curves were presented. Power curves data were obtained multiplying voltage and current. Current density and power density were referred to the projected area of the cathode or of AEM and CEM (7 cm^2^).

## 3 Results and discussion

### 3.1 Membranes characterization

**Fig. 2:**
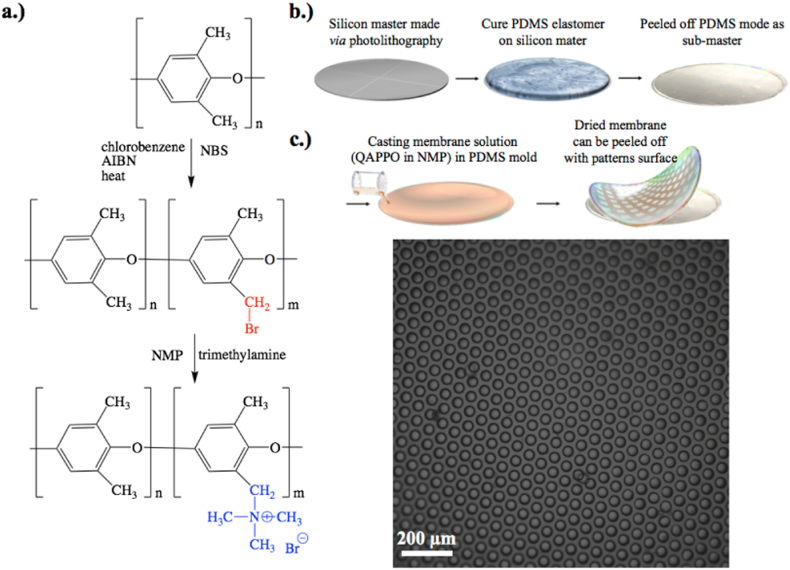
Scheme to synthesize of QAPPO AEMs (a); process flow scheme to make micropatterned QAPPO AEMs (b); and optical micrograph of QAPPO AEM with 40 μm lateral features (c).

Fig. 2.a shows the scheme to synthesize QAPPO AEMs via free radical bromination of commercially available PPO. Fig. 2.b is the process flow scheme to make reusable, micropatterned PDMS molds for fabricating QAPPO AEMs with micropatterned well surfaces. Fig. 2.c is an optical micrograph of a QAPPO AEM with 40 μm lateral features periodically spaced across the membrane surface.

**Fig. 3:**
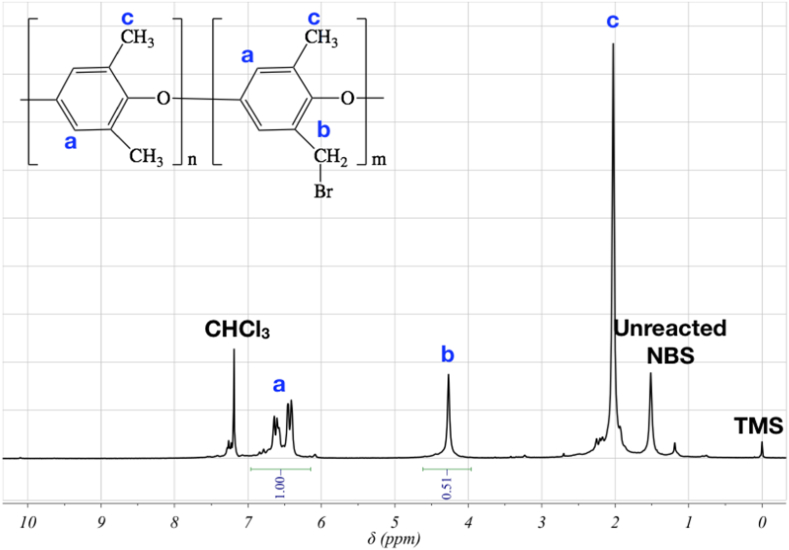
^1^H NMR spectrum of BrPPO.

Fig. 3 is the ^1^H NMR spectra of BrPPO. The signal at 4.5 ppm corresponds to the methylene moiety in the bromomethyl groups. The degree of functionalization (DF) value of the BrPPO is 0.51 (the fraction of repeat units with bromomethyl groups). The anion exchange groups, quaternary benzyl trimethylammonium groups, in PPO are formed by nucleophilic substitution of trimethylamine with the bromine moiety in the bromomethyl groups. For this study, QAPPO AEMs with a low ion-exchange capacity (IEC) (approx. 1.38 mmol g^− 1^) were prepared. The estimated IEC was calculated by the amount of trimethylamine added to the reaction with BrPPO and the DF value of BrPPO [40]. During the drop casting procedure, the unreacted bromomethyl groups self-crosslinked making the QAPPO AEMs insoluble for ^1^H NMR analysis [38].

Table 1 reports the bulk, in-plane chloride ion conductivity of the non-patterned QAPPO AEMs. Because the chemistry of the patterned and non-patterned QAPPO AEMs is the same, no difference in ionic conductivity was expected.

**Table 1:** Chloride ion conductivity of QAPPO Q1 AEM in deionized water and saline solutions.

	σ in DI H_2_O	σ in 5 ppm NaCl	σ in 50 ppm NaCl
QAPPO AEM in the chloride form	0.6 mS cm^− 1^	5.0 mS cm^− 1^	32.9 mS cm^− 1^

### 3.2 Power curves

**Fig. 4:**
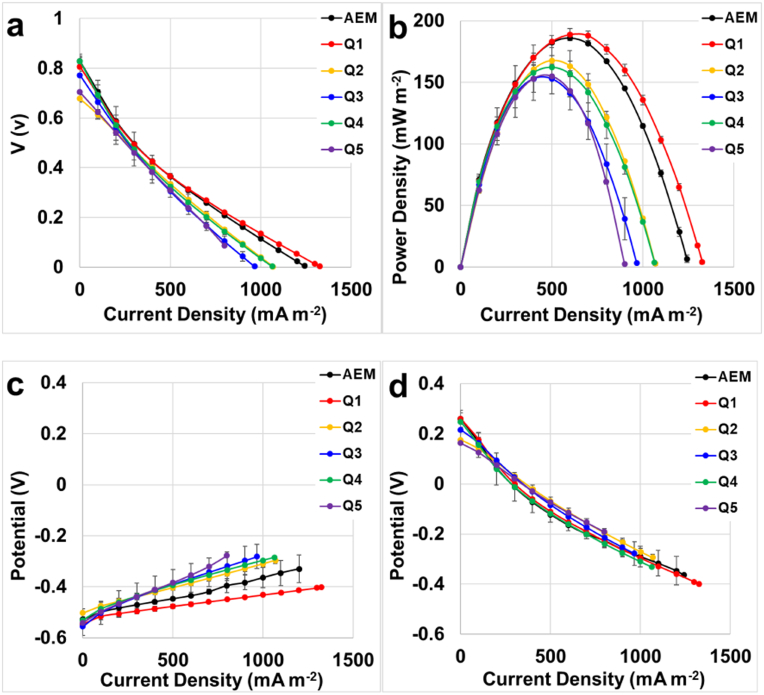
Overall polarization curve (a), power curves (b), anode (c) and cathode (d) polarization curves of the MDCs having different AEM.

Polarization curves (Fig. 4.a), power curves (Fig. 4.b), and anode (Fig. 4.c) and cathode (Fig. 4.d) polarization curves were obtained for the MDCs having different AEMs. The only variable for these experiments was the type of AEM selected. As mentioned above, the electrochemical performance of the MDCs with different AEMs were acquired with initial fresh solutions, while the same and identical anode and cathode electrode were used.

The overall polarization curve (Fig. 4.a) showed initial similar open circuit voltage (OCV) for all the membranes, with an average initial point of 0.75 ± 0.05 V, following similar trends for all the membranes investigated. Although, they had similar voltages at low current, higher voltages for AEM and Q1 were acquired at high current. Near to the short circuit, instead of the straight trend for Q2, Q3, Q4, and Q5; Q1 and the commercial membrane showed a slight different shape recording the maximum current densities at short circuit with 1325 mA m^− 2^ for Q1 and 1242 mA m^− 2^ for the commercial membrane AEM. Polarization curves displayed a linear trend indicating that the cell was largely governed by ohmic overpotentials (see Fig. 4.a).

Power curves were then obtained from the polarization curve. MDC with Q1 membrane recorded the maximum power density of 189 ± 5 mW m^− 2^ at a current density of 600 mA m^− 2^. The commercial membrane obtained slightly lower power generation (186 ± 0.1 mW m^− 2^). MDC with membranes Q2, Q3, Q4 and Q5 lower maximum points of power density recorded. Particularly, the power obtained were 167 ± 4, 153 ± 11, 162 ± 12 and 155 ± 5 mW m^− 2^ respectively. Therefore, MDC with Q1 outperformed the power obtained by the MDC with commercial membrane. The addition of topographical patterns on one side of the membrane did not give any advantage.

The anode (Fig. 4.c) and cathode (Fig. 4.d) polarization curves were taken by inserting a reference electrode within the central chamber that was separated from the anode through the AEM and from the cathode through a CEM. The analysis of the polarization behavior of each electrode agreed with the overall polarization of the cell and their corresponding power curves trends. The polarization behavior of the anode (Fig. 4.c) displayed linear trends in all cases, but with different slopes. The smaller the slope value corresponded with a smaller resistance that was associated with the type of membrane utilized. The results demonstrated that Q1 had the best performances followed by the commercial membrane and by Q2, Q4, Q3 and Q5 respectively. As the anodes utilized were identical in geometrical size and biofilm maturation, the different behavior was solely attributed to the membrane. With respect to the cathode overpotential (Fig. 4.d), the polarization was relatively similar as the same electrode and membrane were used. Examining the cathode and anode polarization behavior together with the overall cell polarization demonstrates that the AEM resistance had a significant impact on power density and cell efficiency.

### 3.3 Desalination

**Fig. 5:**
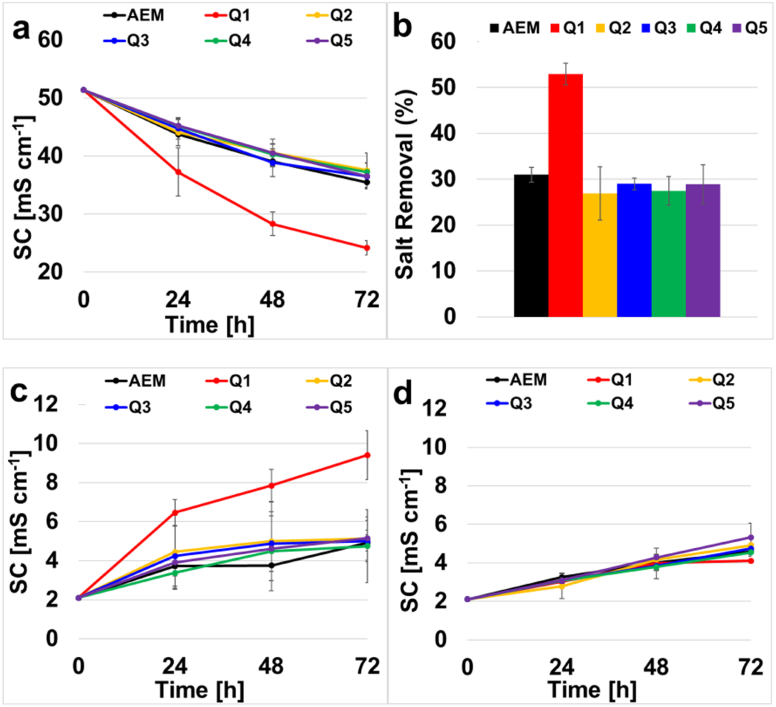
Desalination chamber solution conductivity (a), desalination chamber salt removal (b), anode chamber solution conductivity (c), cathode chamber solution conductivity (d).

The seawater conductivity and pH in the middle chamber of the MDC was 51.4 mS cm^− 1^ and 7.8, respectively. Membrane Q1 provided the greatest removal of salt from the seawater (53 ± 2.7%) and the solution conductivity at the end of the salt removal was 24.2 ± 1.2 mS cm^− 1^. The patterned AEMs (Q2 to Q5) had roughly the same salt removal rate and drop in solution conductivity for the middle chamber in the MDC (see Fig. 5.a and b). Hence, the micropatterned features did not seem to improve the desalination for the MDC. Furthermore, the non-patterned QAPPO AEM had the best performance signaling that the patterned membranes do not enhance desalination. One possible explanation for the lack of added benefit for the patterned membranes is that they may be more prone to fouling. The motivation to use such patterned AEMs was to increase the interfacial area between the solution and the membrane to minimize interfacial resistance.

The solution conductivity of the anode and cathode chamber is given in Fig. 5.c and d. The solution conductivity for both the anode and cathode slightly increased because of the salt removal from the middle, desalination chamber through the ion-exchange membranes. The initial solution conductivity of the anode chamber (Fig. 5.c) was 2.1 mS cm^− 1^ and the final ionic conductivity, for all AEMs besides Q1 (i.e., Q2, Q3, Q4, Q5 and the commercial AEM) was 5.1 mS cm^− 1^. The final solution conductivity of the anode chamber featuring Q1 was 9.4 ± 1.3 mS cm^− 1^, which was higher than the other membranes and corresponded to the greater desalination of the middle chamber.

The cathode chamber ionic conductivity (Fig. 5.d), showed an increase from an initial of 2.1 mS cm^− 1^, to a final value of 4.5 ± 0.5 mS cm^− 1^ for all MDC experiments. The similar values observed were ascribed to the limitation that the same cation exchange membrane was used in all MDC experiments.

### 3.4 pH variation

**Fig. 6:**
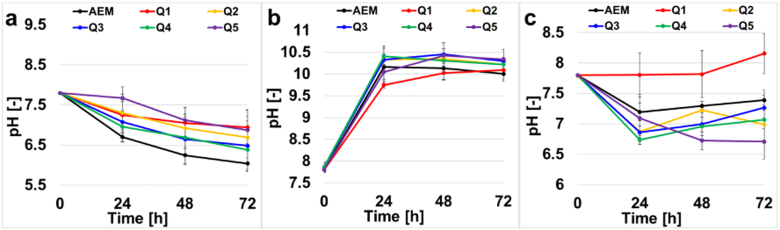
Anode chamber pH (a), cathode chamber pH (b), desalination chamber pH (c).

The pH of the anode chamber (Fig. 6.a) decreased from an initial value of 7.8, to a final range of 6.0 to 6.8 measured at the end of MDC operation. This was due to the oxidation of organics and the production of H^+^ as part of the final products therefore the anodic chamber tends to acidify. Charge neutrality is maintained due to the transport of chloride ions from the middle desalination chamber to the anode. The initial pH in the anode containing activated sludge was always 7.8. The pH of the cathode chamber increased from 7.8 up to 10 in every MDCs (Fig. 6.b). This shift in pH was due to the oxygen reduction reaction (ORR). In fact, the ORR can follow two different patterns: i) acidic with consumption of H^+^ and production of water; or ii) alkaline with production of OH^−^. It is not clear yet which ORR pattern is followed in neutral media but if the acidic way is preferred, H^+^ is consumed from the solution and therefore an abundance of OH^−^ is generated. On the contrary, if the alkaline pattern is followed, the final product is OH^−^. Both ORR patterns can explain the alkalization of the cathode chamber over time.

In order to maintain charge neutrality, sodium ions moves from the desalination chamber to the cathode chamber through the cation exchange membrane. The initial pH for cathode was 7.8 and it increased to a value of 10 at the end of the MDC runs. With respect to the desalination chamber (Fig. 6.c) the pH remained relatively the same or slightly decreased probably because no electrochemical reactions with production/consumption of H^+^ and OH^−^ are occurring.

## 4 Outlook

In this study, different anion exchange membranes were prepared and tested into microbial desalination cells. The results showed that the quaternary ammonium poly(2,6-dimethyl 1,4-phenylene oxide) (QAPPO) anion exchange membranes (AEMs) without topographically patterned surfaces had the highest desalination rate and the best electrochemical performances among the AEMs investigated. Considering the power generation obtained, the current results are lower compared to existing literature [29]. This is mainly due to the operating conditions that in the current case were unfavorable but closer to a real situation rather than a lab-scale condition. The performances as both power generation and desalination rate can be used as baseline to describe the real potential of this technology. In this investigation real seawater and activated sludge rather than sodium chloride containing solution and synthetic wastewater were utilized. The electrolytes choice was dictated in order to simulate the capability of the system to operate in real conditions. In fact, it is well known that high solution conductivity led to higher performances due to the decrease in ohmic losses of the electrolyte [41], [42]. Unfortunately, the activated sludge used in this investigation has a very low solution conductivity (2.1 mS cm^− 1^) that negatively affects the performances. Moreover, the operations were conducted at room temperature (22 ± 2 °C) that is another important factor limiting the anodic kinetics [43], [44]. At last, oxygen was preferred to potassium ferricyanide as oxidant at the cathode in order to mimic real world conditions for applications.

In parallel, the high ions concentration difference between desalination chamber and anode/cathode chamber can enhance other transport phenomena such as diffusion and forward osmosis through the selective membrane leading to a faster desalination rate. The maximum desalination achieved in this study was roughly above 50%, therefore a considerable salt removal was observed. However, the content of salts remaining in solution is still elevated, estimated in approximately 15–16 g L^− 1^, indicating that the water is not suitable for drinking purposes. Present literature considers MDCs suitable devices for reducing salt content before the utilization of the water in reverse osmosis systems [45]. If only MDCs should be used to generate drinking water, we envision the system to be composed by several MDCs hydraulically connected in series in which the solution of the desalination chamber of the first MDC is then inserted into the second MDC, and so on, until 0.3–0.4 g L^− 1^ of salt is achieved. The results from this study show that membrane properties affect the performance of MDCs. Thus, a greater effort should be dedicated for the investigation of selective membranes optimizing membrane thickness, ionic conductivity, and swelling to minimize the ohmic resistances of the MDC to boost power output and desalination rate. Finally, long term performance should be studied to investigate membrane fouling and the possibility of regeneration after washing.

## 5 Conclusions

Non-patterned QAPPO AEMs enhanced the power density and desalination rate for microbial desalination cells when benchmarked against a commercially available AEM. The better performance was ascribed to the lower electrical resistance (i.e., higher ionic conductivity) of the QAPPO AEM due to the fact that the membrane was thinner - translating to a smaller area specific resistance. Although micropatterned QAPPO AEMs can enhance the interfacial area between the solution and membrane to lower interfacial impedance, these patterned membranes are more prone to fouling compromising MDC performance offering no added benefit.
